# Hydroxysafflor Yellow A Attenuates the Apoptosis of Peripheral Blood CD4^+^ T Lymphocytes in a Murine Model of Sepsis

**DOI:** 10.3389/fphar.2017.00613

**Published:** 2017-09-06

**Authors:** Jinping Wang, Ping Wang, Shuiqing Gui, Yun Li, Runhua Chen, Renqing Zeng, Peiyan Zhao, Hanwei Wu, Zheyu Huang, Jianlong Wu

**Affiliations:** ^1^Department of Pharmacy, Shenzhen Second People’s Hospital Shenzhen, China; ^2^Department of Pharmacology and Toxicology, Shenzhen Institute for Drug Control Shenzhen, China; ^3^Department of Intensive Care Unit, Shenzhen Second People’s Hospital Shenzhen, China; ^4^The Central Laboratory, Shenzhen Second People’s Hospital Shenzhen, China

**Keywords:** hydroxysafflor yellow A, sepsis, CD4^+^ T lymphocytes, apoptosis, caspase

## Abstract

Sepsis is generally considered as a severe condition of inflammation that leads to lymphocyte apoptosis and multiple organ dysfunction. Hydroxysafflor yellow A (HSYA) exerts anti-inflammatory and anti-apoptotic effects in infectious diseases. However, the therapeutic effect of HSYA on polymicrobial sepsis remains unknown. This study was undertaken to investigate the therapeutic effects and the mechanisms of action of HSYA on immunosuppression in a murine model of sepsis induced by cecal ligation and puncture (CLP). NIH mice were randomly divided into four groups: control group, sham group, CLP group, and CLP+HSYA group. HSYA (120 mg/kg) was intravenously injected into experimental mice at 12 h before CLP, concurrent with CLP and 12 h after CLP. The levels of circulating inflammatory cytokines, the apoptosis of CD4^+^ and CD8^+^ T lymphocytes, and protein expression of cytochrome C (Cytc), Bax, Bcl-2, cleaved caspase-9, and cleaved caspase-3 were examined. Plasma levels of IL-6, IL-10 and TNF-alpha as well as the apoptosis of CD4^+^ T lymphocytes were increased compared with sham group. These changes were accompanied by increases of pro-apoptotic proteins including Cytc, Bax, cleaved caspase-9, and cleaved caspase-3 and decreases of anti-apoptotic protein Bcl-2 in CD4^+^ T lymphocytes from mice undergoing CLP. In contrast, we fail to observe significant effect of HSYA on the apoptosis of CD8^+^ T lymphocytes in CLP-treated group. Of note, HSYA treatment reversed all above changes observed in CD4^+^ T lymphocytes, and significantly increased the ratio of CD4^+^:CD8^+^ T lymphocytes in CLP-treated mice. In conclusion, HSYA was an effective therapeutic agent in ameliorating sepsis-induced apoptosis of CD4^+^ T lymphocytes probably through its anti-inflammatory and anti-apoptotic effects.

## Introduction

Sepsis is a life-threatening condition caused by dysregulated host response to infection (Singer et al., 2016; Thompson et al., 2017), and has been recognized as one of the most significant causes for mortality worldwide (Fullerton et al., 2017; Gunes Ozaydin et al., 2017). The incidence and the absolute number of deaths are continuing to rise rapidly (Suarez De La Rica et al., 2016). Although significant advances have been achieved in intensive care treatment and in organ supporting, treatment of sepsis remains challenging in critical care medicine (Kaukonen et al., 2014; Seeley and Bernard, 2016). The pathophysiology of sepsis is characterized by marked immunosuppression (Boomer et al., 2011), and immune cell apoptosis is thought to be a major contributor to this immunosuppressive status under septic conditions (Chen H.M. et al., 2016; Sonego et al., 2016). Emerging evidence suggests that peripheral CD4^+^ T lymphocytes undergo apoptosis in response to septic conditions, leading to marked decreases in numbers of CD4^+^ T lymphocytes (Cabrera-Perez et al., 2014; Gao et al., 2016). Therapies that prevent immune cell apoptosis could significantly reduce the mortality rate of sepsis (Oberholzer et al., 2001). Mitochondria dysfunction plays a key role in promoting apoptosis in T cells (Liesa and Shirihai, 2016; Uzhachenko et al., 2016), and inhibition of the mitochondria-dependent apoptotic pathway is essential for the development and maintenance of the immune system (Chikka et al., 2016). Moreover, mitochondrial dysfunction and decreased expression of mitochondrial proteins occur during sepsis. Hence, impaired mitochondrial activity has been proposed as an important causative factor for sepsis (Duran-Bedolla et al., 2014; Zheng et al., 2015). Thus, agents that protect mitochondria dysfunction may improve the clinical outcomes of septic patients.

Xuebijing (XBJ) is a traditional Tibetan medicine, which has been widely applied to treat systemic inflammatory response syndrome (SIRS), such as sepsis and associated multiple organ dysfunction syndrome (MODS) (Wang et al., 2015; Chen S. et al., 2016). XBJ is the only proprietary Chinese medicine that has been approved for the treatment of sepsis by the State Food and Drug Administration of China (Huang et al., 2011; Fang and Wang, 2013). However, the underlying mechanism of XBJ’s actions has not yet been well characterized. As an important bioactive compound isolated from XBJ, Hydroxysafflor yellow A (HSYA) has been demonstrated to possess multiple bio-activities, including scavenging reactive oxygen species, inhibition of mitogen-activated protein kinase activation, and alteration of inflammatory cytokine expression (Chen et al., 2013; Jiang et al., 2014). HSYA is protective against lipopolysaccharide-induced acute lung injury in mice (Liu et al., 2014). However, whether HSYA has therapeutic effects on experimental sepsis remains to be determined.

In this study, we applied a widely used mouse model of sepsis induced by cecal ligation and puncture (CLP) and investigated the therapeutic effects of HSYA on sepsis as well as its impact on peripheral blood CD4^+^ T lymphocytes apoptosis and the modulation of apoptosis-associated proteins Bax, Bcl-2, cleaved caspase-9, cleaved and caspase-3.

## Materials and Methods

### Animals

The Laboratory Animal Care and Use Committee of Shenzhen Second People’s Hospital approved all animal related experimental protocols. Seventy-two male NIH mice (10- to 12-weeks-old; 20–25 g, obtained from Center of Experimental Animals of NanFang Medical University, Guangzhou, China) were acclimatized under controlled conditions for a week before surgical operations, and housed in cages located in temperature-controlled rooms with a 12 h/12 h light-dark cycle. The mice received standard laboratory chow and tap water *ad libitum*.

### CLP Model of Sepsis

Cecal ligation and puncture mouse model of sepsis was established according to a previously published protocol (Li et al., 2010). Briefly, mice were anesthetized with pentobarbital (50 mg/kg) by intraperitoneal injection. A midline incision (approximately 1 cm) was made in the abdominal wall to exteriorize the cecum. The cecum was then ligated 0.5 cm from the apex using a 7-0 silk suture. The distal cecum was punctured with a sterile 18-gauge needle. A small amount of luminal contents was extruded into the peritoneal cavity. After the cecum was returned to the abdominal cavity, the abdominal muscle and skin incisions were closed. As a control, sham-operated mice underwent the same procedure, including peritoneal opening and bowel exposure, but without ligation or perforation of the cecum. After surgery, all mice received fluid resuscitation via the subcutaneous injection of normal saline (1 mL). All mice have free access to food and water after recovery from anesthesia.

### Experimental Design and Sample Collection

**FIGURE 1:**
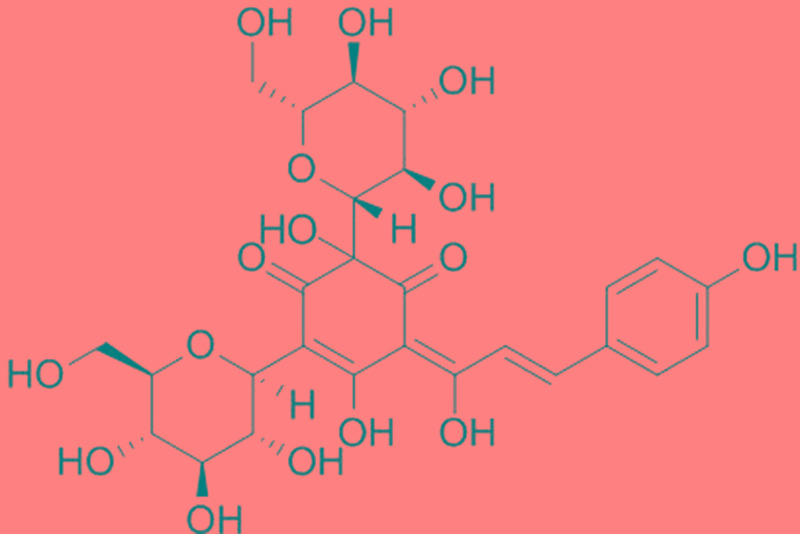
The chemical structure of hydroxysafflor yellow A (HSYA).

Hydroxysafflor yellow A (purity > 98% by HPLC, the chemical structure is shown in **Figure 1**) was purchased from the National Institutes for Food and Drug Control (Beijing, China), and dissolved in normal saline. Mice were randomly divided into four groups (*n* = 18): (1) control group in which mice received normal saline; (2) sham group in which mice underwent sham operation and received normal saline; (3) CLP group in which mice were subjected to CLP and received normal saline; (4) HSYA+CLP group in which mice were subjected to CLP and pretreated with HSYA (120 mg/kg) 12 h before the operation, and at 0 and 12 h after CLP. Plasma and peripheral blood were harvested 24 h after surgery as described below.

Mice in each group were divided into three subgroups (*n* = 6 per group). Each subgroup was used for different experiment; i.e., ELISA measurement for cytokines, flow cytometry analysis of apoptosis in lymphocytes, and Western blotting of apoptosis associated proteins. Peripheral blood was collected from orbital sinus.

### Measurement of Cytokines

Serum concentrations of TNF-alpha, IL-6, and IL-10 were measured using a murine ELISA kit according to the manufacturer’s protocols (eBioscience Co., Ltd., San Diego, CA, United States).

### Detection of Apoptosis

Heparinized peripheral blood samples were collected. Briefly, the erythrocytes were lysed with ammonium chloride (150 mM) and washed twice in phosphate-buffered saline. The suspended cells were incubated for 15 min at 4°C with annexin V-FITC, PI, fluorescently tagged anti-mouse CD3^+^ and CD45^+^ antibodies for analysis of T lymphocytes apoptosis. Stained cells were detected using a FACSCalibur flow cytometry instrument. For each sample, 10,000 events were recorded, and analyzed by CellQuest software (BD Biosciences). First, T lymphocyte population was characterized for each group by CD3^+^/CD45^+^ cells staining. The apoptotic of the T lymphocyte was detected for each group by annexin V-FITC and propidium iodide (PI) staining. The T lymphocytes were stained with fluorescently tagged anti-mouse CD4^+^ and CD8^+^ antibodies. Three-color data were acquired using a FACStarPlus flow cytometer. Data analysis was performed using Attractors and CellQuest software. Each subpopulation was gated using forward and side scatter and fluorescence intensity for the specific CD4^+^ and CD8^+^ markers. Multicolor flow cytometric analysis was used to determine the frequency of apoptotic cells. The amount of early apoptosis and late apoptosis was determined as the percentage of annexin V^+^/PI^-^ and annexin V^+^/PI^+^, respectively. Thus, we estimated the total number of apoptotic cells as the percentage of cells positive for annexin V.

### Purification of Mouse CD4^+^ T Lymphocyte

Blood samples were added gently onto a Histopaque solution, and then centrifuged at 400*g* for 40 min. Subsequently, the phase containing peripheral blood mononuclear cells was transferred to another tube. The cells were then washed twice with RPMI 1640 medium supplemented with 1% antibiotic, and centrifuged at 400*g* for 10 min. CD4^+^ T lymphocytes were purified from peripheral blood using anti-CD4^+^ microbeads on a magnetic sorter. The purity of theCD4^+^ T lymphocytes was >90%.

### Western Blot Analysis

Due to the fact that individual mouse sample was not sufficient for western blot analysis, the CD4^+^ T lymphocytes from each mouse in same group were pooled. Cells were lyzed in lysis buffer supplemented with protease inhibitor cocktail from Sigma (Le et al., 2012). All samples are whole cell lysates except samples for Cytc are cytosolic fractions prepared with a cell fractionation kit from Pierce. Protein concentration was quantified using BCA assay. Then, 5× loading buffer was added and samples were boiled at 100° for 10 min. Equal amount of protein was loaded onto 8–12% SDS–PAGE, and transferred to polyvinylidene fluoride membranes using a semi-dry Trans-Blot transfer cell. After blocking with 5% (w/v) non-fat milk for 1 h at room temperature, the membranes were incubated overnight at 4°C with the primary antibodies and then incubated with anti-mouse or anti-rabbit secondary antibody for 1 h at room temperature. Bands were detected using an enhanced chemiluminescence detection system (iNtRON Biotechnology, Seongnam, South Korea) according to the manufacturer’s instructions. The intensities of the immune-reactive bands were determined using TotalLabTL120 software (Nonlinear Dynamics Ltd., United Kingdom). Primary antibodies against Bax, Bcl-2, cleaved caspase-3, and cleaved caspase-9 and the secondary antibodies were obtained from Santa Cruz Biotechnology.

### Statistical Analysis

All data were presented as the mean ± SD. One-way ANOVA followed by Turkey *post hoc* test was applied to analyze the differences of lymphocytes apoptosis, blood CD4:CD8 T lymphocytes ratio, CD4^+^ T lymphocytes, CD8^+^ T lymphocytes apoptosis, and protein levels of Bcl-2, Cytc, Bax, cleaved caspase-3, and cleaved caspase-9 in CD4^+^ T lymphocytes as well as the differences of IL-6, IL-10, and TNF-alpha. All analyses were conducted by using the Graphpad Prism software version 5.0 (GraphPad Software, Inc.). *P* < 0.05 was considered to be statistically significant.

## Results

To define the optimal dosage of HSYA to be used in this study, we conducted a preliminary experiment and tested three different dosages in mice that were subjected to CLP operation: 60, 120, and 180 mg/kg. All mice survived at the end of the experiments and no noticeable side effects were observed for all three dosages during the 24 h experimental duration (Supplementary Table 1). Our preliminary data showed that compared with the CLP group, the lymphocyte counts and the serum levels of inflammatory cytokines of HSYA-treated mice were significantly improved with a daily dosage of 120 mg/kg or higher (Supplementary Figure 1 and Table 2). Since 120 mg/kg HSYA has been previously reported to be protective in lipopolysaccharide-induced acute lung injury (Liu et al., 2014), we finally chose the median dose of 120 mg/kg in this study.

### Effects of HSYA on Serum Cytokine Levels in Septic Mice

**FIGURE 2:**
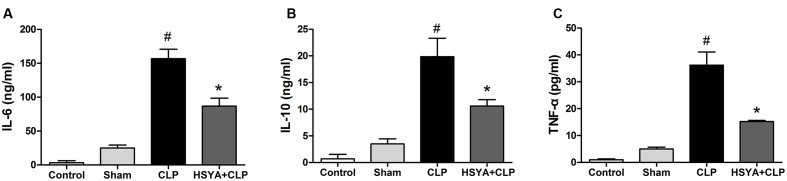
Effects of HSYA on the levels of cytokines in peripheral blood. HSYA (120 mg/kg) was intravenously injected at 12 h before the operation, and 0 and 12 h after CLP operation. Serum levels of **(A)** IL-6 **(B)** IL-10 and **(C)** TNF-alpha were detected by ELISA 24 h after CLP. Data are presented as mean ± SD of 6 animals per group. ^#^Denotes significant differences (*P* < 0.05) compared to control group. ^∗^Denotes significant differences (*P* < 0.05) compared to CLP group.

Inflammatory cytokines play a central role in the pathogenesis of sepsis. Higher levels of cytokines (IL-6, IL-10, etc.) have been shown to be associated with diseases severity. The measurement of serum cytokines was useful in determining the inflammation status of the mice. The concentrations of IL-6, IL-10, and TNF-alpha in the serum of the CLP group were156.52 ± 13.85 ng/mL, 19.85 ± 2.13 ng/mL, and 36.2 ± 5.4 pg/mL, respectively, which were significantly higher than that of the control group or sham group (**Figure 2**). The levels of cytokines in the HSYA+CLP group were significantly reduced to 86.75 ± 11.85 ng/mL, 10.59 ± 1.19 ng/mL, and 15.20 ± 0.41 pg/mL for IL-10, IL-6, and TNF-alpha, respectively. Of note, HSYA treatment led to a 1.80-, 1.90-, and 2.28-fold reduction in the concentrations of IL-6, IL-10, and TNF-alpha, respectively, compared to the CLP group (*P* < 0.05).

### Effects of HSYA on Peripheral T lymphocyte Apoptosis in Septic Mice

**FIGURE 3:**
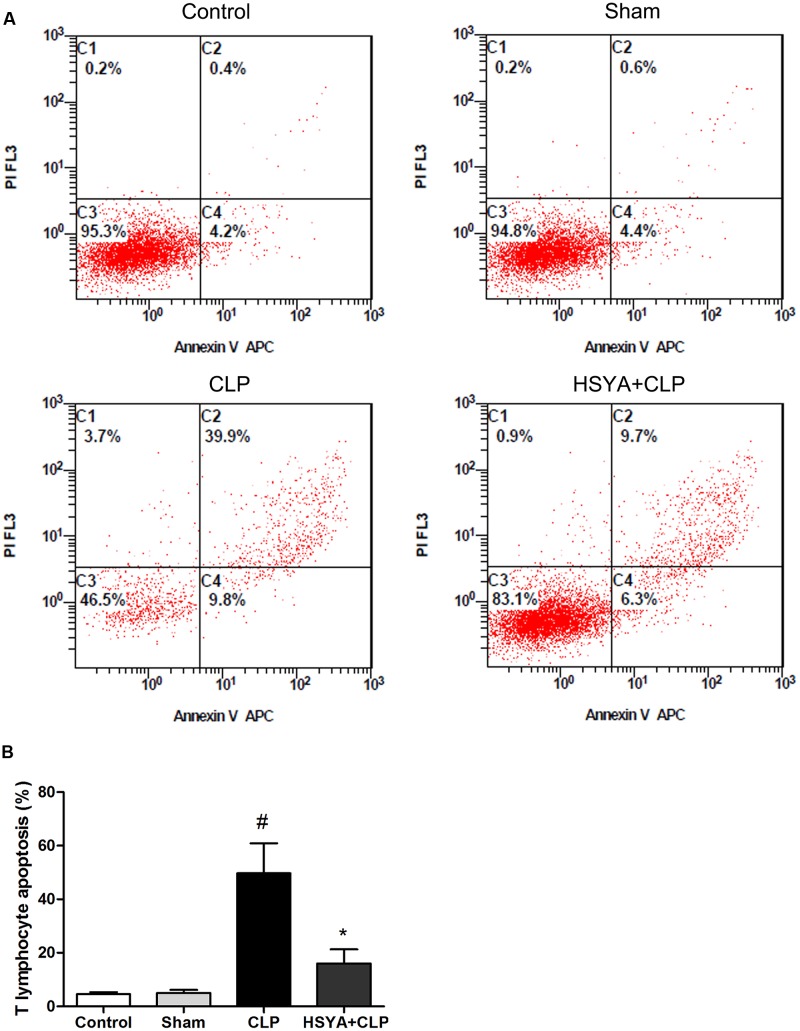
Effects of HSYA on the apoptosis of peripheral blood T lymphocytes. HSYA (120 mg/kg) was intravenously injected at 12 h before the operation, and 0 and 12 h after CLP operation. **(A)** Annexin V-FITC and propidium iodide (PI) staining was used to detect T lymphocyte apoptosis in peripheral blood, **(B)** analysis of T lymphocyte apoptosis among all experiment groups. T lymphocyte population was characterized by CD3^+^/CD45^+^ cells staining. The apoptotic of the T lymphocyte were labeled with fluorescent tagged Annexin V (FITC) and PI and subjected to flow cytometry analysis at 24 h after CLP. The cells positive for annexin V were determined to be apoptotic cells. Data are presented as mean ± SD of 6 animals per group. ^#^Denotes significant differences (*P* < 0.05) compared to control group. ^∗^Denotes significant differences (*P* < 0.05) compared to CLP group.

Cecal ligation and puncture-induced immunosuppression is characterized by increased apoptosis of lymphocytes. T lymphocyte apoptosis has been increasingly recognized as an important step in the pathogenesis of experimental sepsis. T lymphocyte population was characterized by CD3^+^/CD45^+^ cells staining. The cells positive for annexin V were determined to be apoptotic cells. Twenty-four hours after surgery, the percentage of apoptotic peripheral T lymphocytes was significantly increased in the CLP group (49.7 ± 11.2%, *P* < 0.05), but not in sham-operated group (5.0 ± 1.2%, *P* > 0.05), compared with the control group (4.6 ± 0.7%). HSYA treatment significantly decreased CLP-induced peripheral lymphocyte apoptosis (16.0 ± 5.3% versus 49.7 ± 11.2%, *P* < 0.05) (**Figure 3** and Supplementary Figure 2).

### Effects of HSYA on the Ratio of Peripheral CD4^+^:CD8^+^ T Lymphocytes

**FIGURE 4:**
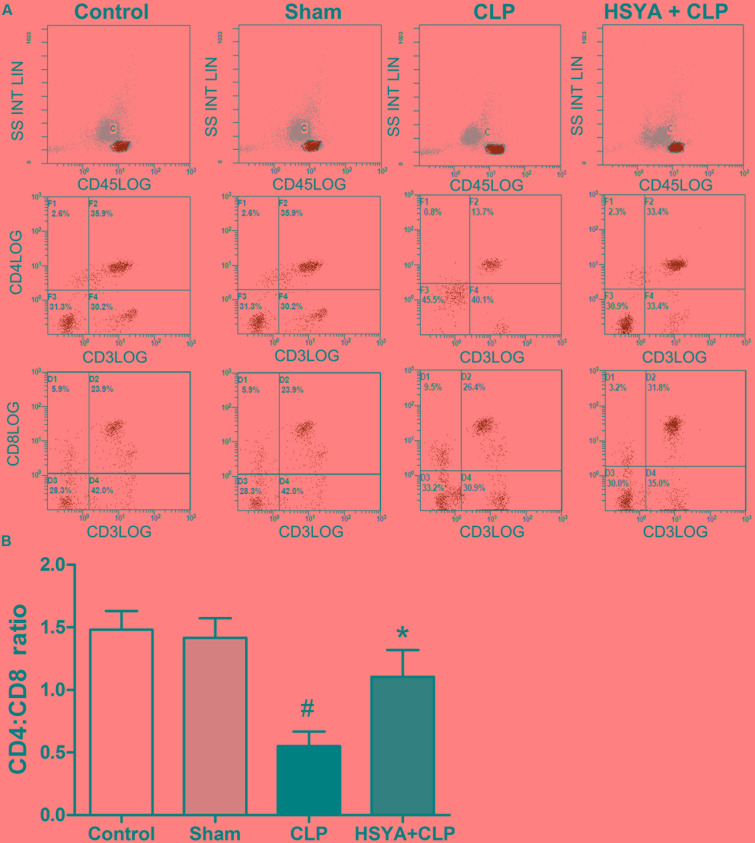
Effects of HSYA on peripheral blood CD4^+^:CD8^+^ T lymphocyte ratio. HSYA (120 mg/kg) was intravenously injected at 12 h before the operation, and 0 and 12 h after CLP operation. **(A)** Percentage of CD4^+^ T lymphocytes and CD8^+^ T lymphocytes in peripheral blood T lymphocytes among all experiment groups, **(B)** analysis of the Ratio of Peripheral CD4^+^:CD8^+^T lymphocytes among all experiment groups. The T lymphocytes were stained with fluorescently tagged anti-mouse CD4^+^ and CD8^+^ antibodies. Three-color data were acquired using a FACStarPlus flow cytometer. Data are presented as the mean ± SD of 6 animals per group. ^#^Denotes significant differences (*P* < 0.05) compared to control group. ^∗^Denotes significant differences (*P* < 0.05) compared to CLP group.

Phenotypic analysis of CD4^+^ and CD8^+^ T lymphocytes revealed an inverted ratio of CD4^+^:CD8^+^ T lymphocytes following septic injury in mice. The CD4^+^:CD8^+^ T lymphocyte ratio was significantly decreased in the CLP group (0.52 ± 0.16, *P* < 0.05), but not in the sham group (1.48 ± 0.05, *P* > 0.05) groups, compared to the control group (1.5 ± 0.12%). HSYA treatment significantly increased the CD4^+^:CD8^+^ T lymphocyte ratio (1.05 ± 0.19) compared to the CLP group (*P* < 0.05) (**Figure 4**).

### Effects of HSYA on Peripheral CD4^+^ and CD8^+^ T Lymphocyte Apoptosis in Septic Mice

**FIGURE 5:**
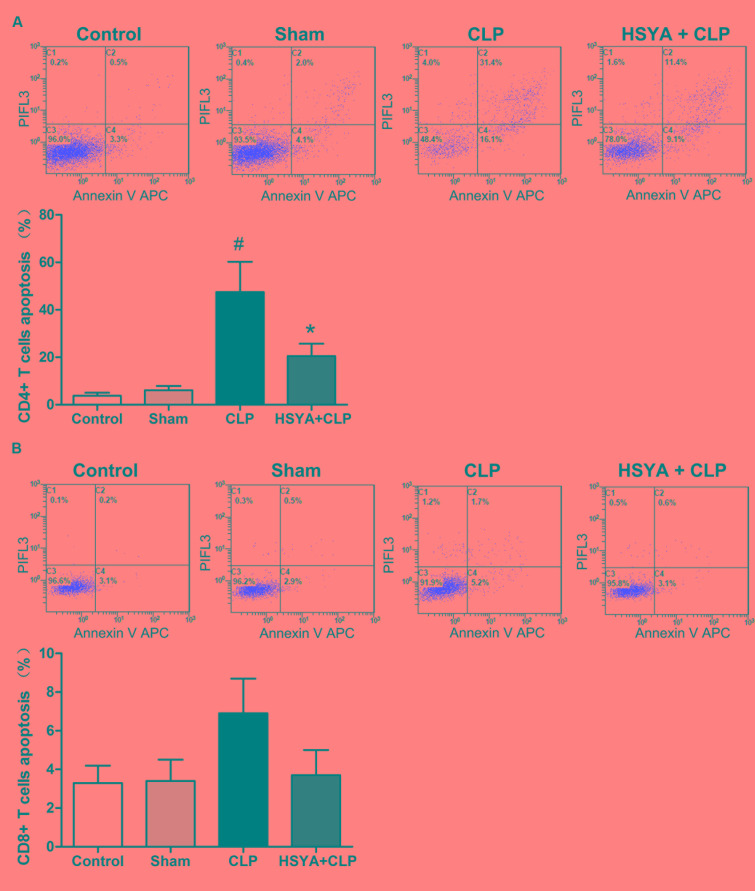
Effects of HSYA on the apoptosis of peripheral blood CD4^+^ T lymphocytes, CD8^+^ T lymphocytes apoptosis. **(A)** CD4^+^ T lymphocytes apoptosis, **(B)** CD8^+^ T lymphocytes apoptosis. HSYA (120 mg/kg) was intravenously injected at 12 h before the operation, and 0 and 12 h after CLP operation. The T lymphocytes were stained with fluorescently tagged anti-mouse CD4^+^ and CD8^+^ antibodies. Each subpopulation was gated using forward and side scatter and fluorescence intensity for the specific CD4^+^ and CD8^+^ markers. Multicolor flow cytometric analysis was used to determine the percentage of apoptotic cells. The cells positive for annexin V were determined to be apoptotic cells. Data are presented as the mean ± SD of 6 animals per group. ^#^Denotes significant differences (*P* < 0.05) compared to control group. ^∗^Denotes significant differences (*P* < 0.01) compared to CLP group.

Within the immune system, T lymphocytes are divided into conventional CD4 and CD8 populations, CD4^+^ T lymphocyte is important players in the proper development of numerous cellular and humoral immune responses. CD8^+^ T lymphocytes are important in both the clearance of infection and the generation of memory. Annexin V staining was applied to assess CD4^+^ and CD8^+^ T lymphocyte apoptosis. As shown in **Figure 5**, mice in the CLP group showed a large percentage (47.5 ± 12.7%) of annexin V positive CD4^+^ T lymphocytes, which was about 11-fold higher than the control group (3.8 ± 1.2%), while the sham group showed a less than onefold increase (6.1 ± 1.8%) compared to the control group (**Figure 5A**). HSYA significantly attenuated the increase of CD4^+^ T lymphocytes apoptosis in CLP mice (20.5 ± 5.2% versus 47.5 ± 12.7%, *P* < 0.05) (**Figure 5A**).

Similar to CD4^+^ T lymphocytes, the percentage of apoptotic CD8^+^ T lymphocytes was also increased in the CLP group (6.9 ± 1.8%), but not in the sham group (3.4 ± 1.1%), compared to the control group (3.3 ± 0.9%). Of note, the increase in CD8^+^ T lymphocytes apoptosis was less (∼1-fold increase) than that of CD4^+^ T lymphocytes (∼11-fold increase). Most likely, as substantial number of CD4^+^ T lymphocytes that underwent apoptosis accounted for the decreased ratio of CD4^+^:CD8^+^ T lymphocyte in CLP animals (**Figure 5B**). Compared with CLP group, HSYA treatment could inhibit CLP-induced apoptosis in CD8^+^ T lymphocytes (3.7 ± 1.0% versus 6.9 ± 1.8%) (**Figure 5B**). However, no significant differences were observed for CD8^+^ T lymphocyte apoptosis across the groups (*P* > 0.05).

### Anti-apoptotic Mechanisms of HSYA in CD4^+^ T Lymphocytes

**FIGURE 6:**
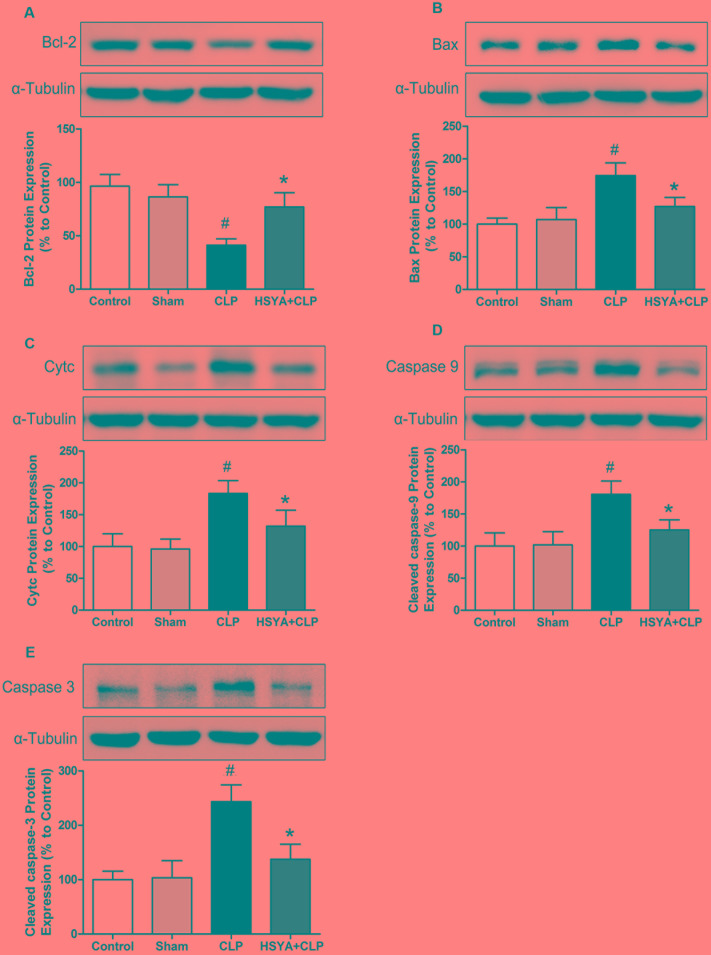
Effects of HSYA on the protein levels of Bcl-2, Cytc, Bax, cleaved caspase-3, and cleaved caspase-9 in CD4^+^ T lymphocytes. HSYA (120 mg/kg) was intravenously injected at 12 h before the operation, and 0 and 12 h after CLP operation. Protein levels of Bcl-2 **(A)**, Bax **(B)**, Cytc **(C)**, cleaved caspase-9 **(D)**, and cleaved caspase-3 **(E)** were determined by western blot using whole cell lysates **(A,C–E)** and cytosolic fractions **(B)** at 24 h after CLP. Alpha-tubulin was used as the loading control. Data are presented as mean ± SD of 6 animals per group. ^#^Denotes significant differences (*P* < 0.05) compared to control group. ^∗^Denotes significant differences (*P* < 0.05) compared to CLP group.

To understand the anti-apoptotic mechanisms of HSYA in CD4^+^ T lymphocytes under septic conditions, we assessed the expression of several apoptosis-associated proteins in these cells. As shown in **Figure 6** and Supplementary Figure 3, the level of the anti-apoptotic protein Bcl-2 was significantly decreased in CD4^+^ T lymphocytes from CLP-treated mice (∼42.67% of the control group, *P* < 0.05), but not that from sham-treated mice (∼89.70% of the control group, *P* > 0.05) (**Figure 6A**). The levels of the pro-apoptotic proteins Bax, CytC, cleaved caspase-9, and cleaved caspase-3 were significantly increased in CD4^+^ T lymphocytes from CLP group, which was 1.75-, 1.83-,1.80-, and 2.43-fold higher than the control group, respectively (*P* < 0.05) (**Figures 6B–E**). No significant changes of these proteins were observed in the sham group (*P* > 0.05, **Figures 6B–E**). HSYA treatment significantly attenuated the decrease of Bcl-2 protein and the increase of Bax, CytC, cleaved caspase-9, and cleaved caspase-3 proteins in CD4^+^T lymphocytes from CLP treated mice (*P* < 0.05).

## Discussion

Sepsis is major clinical challenge with limited therapeutic options in critical care medicine (Liu et al., 2016). Hence, there is an urgent need to identify new therapeutic agents to improve sepsis outcomes. In the present study, the effects of HSYA on murine sepsis induced by CLP were examined. The results indicate that HSYA treatment effectively reduces sepsis-induced apoptosis of peripheral blood CD4^+^ T lymphocytes, and this protection may be associated with its inhibitory action on inflammatory response and its modulation of pro-/anti-apoptotic protein expression, including Cytc, Bax, Bcl-2, cleaved caspase-9, and cleaved caspase-3.

It is generally accepted that uncontrolled inflammation is the main factor that contributes to organ dysfunction and death during sepsis (Boomer et al., 2011; Rossaint and Zarbock, 2015). Studies of genetic polymorphisms have demonstrated higher severity and mortality in septic patients that harbor a genotype expressing higher levels of TNF-alpha, IL-10, and IL-6 (Feng et al., 2015; Giamarellos-Bourboulis and Opal, 2016). Cytokines such as TNF-alpha, IL-10, and IL-6 can activate pro-coagulation factors in the cells lining blood vessels and lead to endothelial damage. The damaged endothelial surface inhibits anticoagulant properties as well as increases anti-fibrinolysis, leading to a SIRS, sepsis shock, and MODS. In the present study, we observed that the production of IL-6, IL-10, and TNF-alpha was increased after CLP, and this increase was significantly attenuated by HSYA treatment. Our results indicate that HSYA can reduce the release of inflammatory cytokines under septic conditions. Previously, Li et al. (2016) have also shown that safflower yellow significantly reduces 28-day mortality and increases survival in patients with severe sepsis and septic shock, and decreasing inflammatory reaction accounts for this protection.

Previous studies have also shown that the apoptosis of T lymphocytes is closely associated with a fatal outcome in patients with sepsis (Lin et al., 2014; Gao et al., 2015). Consistent with these findings, in the present study, we found that peripheral T lymphocyte apoptosis was significantly higher in septic mice than the control mice, and HSYA treatment significantly reduces sepsis-induced CD4^+^ T lymphocyte apoptosis.

CD4^+^ T lymphocytes are the main effector cells in the adaptive immune system. A number of CD4^+^ T lymphocytes are exhausted by the numerous inflammatory mediators which are released during sepsis (Shao et al., 2015; Tian et al., 2015). Consistent with previous studies, our data show that CD4^+^ T lymphocytes number decrease in septic mice, and this decrease is significantly attenuated by HSYA treatment. Unlike CD4^+^ T lymphocytes sepsis induces a slight increase (which was not statistically significant) in CD8^+^ T lymphocytes apoptosis under our experimental conditions. As a consequence, a larger population of CD4^+^ T lymphocytes undergoes apoptosis than CD8^+^ T lymphocytes, which leads to a decreased ratio of CD4^+^:CD8^+^ T lymphocytes in the peripheral blood and accounts for the immunosuppressive status in septic mice. HSYA treatment appears to improve sepsis-induced immunosuppression through inhibiting CD4^+^ T lymphocyte apoptosis.

A large body of evidence has shown that mitochondrial dysfunction plays an important role in sepsis-mediated multi-organ damage. Mitochondrial alteration is one of the main pathways that regulate Bcl family proteins and caspase-independent apoptosis (Parlato et al., 2014; Birkinshaw and Czabotar, 2017). The Bcl family includes pro- and anti-apoptotic proteins, whose interactions regulate cell fate. The pro-apoptotic members of the Bcl family transmit apoptotic stimuli by activating Bax. The anti-apoptotic members, such as Bcl-2, counteract this process by binding and neutralizing the pro-apoptotic signals. After Bax forms pores on the mitochondrial outer membrane, Cytc is released, which participates in the formation of the apoptosome with caspase-9 and activates caspase-3 (Shamas-Din et al., 2011; Kiraz et al., 2016). Therefore, we hypothesized that HSYA may inhibit CD4^+^ T lymphocyte apoptosis by regulating proteins in intrinsic mitochondrial pathway during sepsis. Our results confirm that HSYA treatment up-regulates the level of Bcl-2 expression, and suppresses the expression of Bax, Cytc, cleaved caspase-3, and cleaved caspase-9. Therefore, a high Bcl-2:Bax ratio indicates resistance to apoptosis, which explains our findings that less CD4^+^ T lymphocyte apoptosis is observed in HSYA-treated septic mice.

Although, our preliminary experiments and previous studies have demonstrated that 120 mg/kg of HSYA is an effective and safe dosage (Liu et al., 2004), it is still a limitation of this study for using single dose of HSYA to determine its protective effects in polymicrobial sepsis. With this caveat, we were unable to determine whether the therapeutic effect is dose-dependent. Furthermore, we have not thoroughly examined the effect of HSYA itself on the population of immune cells in normal mice. Further studies are required to confirm whether HSYA is safe in normal mice at therapeutic doses. Lastly, the duration of the intervention was based on the previous evidence from pharmacokinetic/pharmacodynamic studies in rodents and in humans (Chu et al., 2006; Li et al., 2015). The reason we gave HSYA to mice 12 h before the CLP operation was based on the time of whole distribution process *in vivo*, however, we didn’t measure the plasma levels of HSYA and its metabolites. Despite these limitations, the results of the present study provide novel insights into the clinically relevant therapeutic effects of HSYA on sepsis.

## Conclusion

Our results indicate that HSYA treatment improves sepsis-induced immunosuppression via inhibiting CD4^+^ T lymphocytes apoptosis under septic conditions. Moreover, HSYA exerts its anti-apoptotic effects by upregulating the expression of Bcl-2 protein as well as inhibiting protein expression of Cytc, Bax, cleaved caspase-3, and cleaved caspase-9. In summary, HSYA can be developed as a potential anti-septic drug.

## Author Contributions

JPW and JLW participated in research design. PW, RC, PZ, RZ, and HW conducted experiments. PZ, SG, ZH, and JPW performed data analysis. JPW, PW, and JLW contributed to the writing of the manuscript. All authors have read and approved the final manuscript.

## Conflict of Interest Statement

The authors declare that the research was conducted in the absence of any commercial or financial relationships that could be construed as a potential conflict of interest.
