# Supplementary material for: Hydroxysafflor Yellow A Attenuates the Apoptosis of Peripheral Blood CD4+ T Lymphocytes in a Murine Model of Sepsis
Source: Front Pharmacol. 2017 Sep 6;8:613. doi: 10.3389/fphar.2017.00613 (PMC5592278; doi:10.3389/fphar.2017.00613)
Supplement: Supplementary file 1 [file Table_1.PDF]

Supplementary Table1:Liver and Renal function in each group(mean±SD,N=6)

	ALT (U/L)	AST (U/L)	BUN mmol/L	Creatinine μmol/L
Control	27.13±2.52	56.91±2.72	4.07±0.61	18.83±3.01
Sham	24.34±1.94	60.87±5.57	4.80±0.30	17.97±3.12
CLP	50.56±8.48#	140.23±12.98#	8.30±0.60#	19.93±4.01
HSYA(60mg/kg)+CLP	47.03±5.99	133.56±8.48	7.63±1.14	18.33±4.51
HSYA(120mg/kg)+CLP	33.34±2.33*	90.35±12.40*	6.67±0.60*	17.00±2.01
HSYA(180mg/kg)+CLP	29.67±3.38*	69.01±8.81*	6.10±0.50*	17.60±1.65

Various doses of HSYA (60,120, 180mg/kg) were intravenously injected at 12h before the operation, and 0h and 12hafter CLP operation. Liver and renal function was measured. #Denotes significant differences (P<0.05) compared to control group.*Denotes significant differences (P< 0.05) compared to CLP group. WBC: white blood cell count, NEUT: neutrophilic granulocyte. ALT: alanine transaminase, AST: aspartate transaminase, BUN: blood urea nitrogen.
