# Supplementary material for: Hydroxysafflor Yellow A Attenuates the Apoptosis of Peripheral Blood CD4+ T Lymphocytes in a Murine Model of Sepsis
Source: Front Pharmacol. 2017 Sep 6;8:613. doi: 10.3389/fphar.2017.00613 (PMC5592278; doi:10.3389/fphar.2017.00613)
Supplement: Supplementary file 2 [file Table_2.PDF]

Supplementary Table2:Blood cell count in each group(mean±SD,N=6)

	WBC ($\times 10^9$/L)	Lymphocytes($\times 10^9$/L)	NEUT($\times 10^9$/L)
Control	3.46±0.08	2.94±0.11	0.29±0.06
Sham	3.89±0.13	2.36±0.15	0.22±0.03
CLP	1.23±0.05#	0.60±0.06#	0.06±0.01#
HSYA(60mg/kg)+CLP	1.96±0.38	1.07±0.56	0.08±0.01
HSYA(120mg/kg)+CLP	2.78±0.47*	1.97±0.13*	0.13±0.02*
HSYA(180mg/kg)+CLP	2.91±0.28*	2.10±0.35*	0.18±0.01*

Various doses of HSYA (60,120, 180mg/kg) were intravenously injected at 12h before the operation, and 0h and 12hafter CLP operation. Blood count was measured. # Denotes significant differences ($P<0.05$) compared to control group. *Denotes significant differences ($P< 0.05$) compared to CLP group. WBC: white blood cell count, NEUT: neutrophilic granulocyte.
