# Supplementary material for: Hydroxysafflor Yellow A Attenuates the Apoptosis of Peripheral Blood CD4+ T Lymphocytes in a Murine Model of Sepsis
Source: Front Pharmacol. 2017 Sep 6;8:613. doi: 10.3389/fphar.2017.00613 (PMC5592278; doi:10.3389/fphar.2017.00613)
Supplement: Supplementary file 3 [file Image_1.PDF]

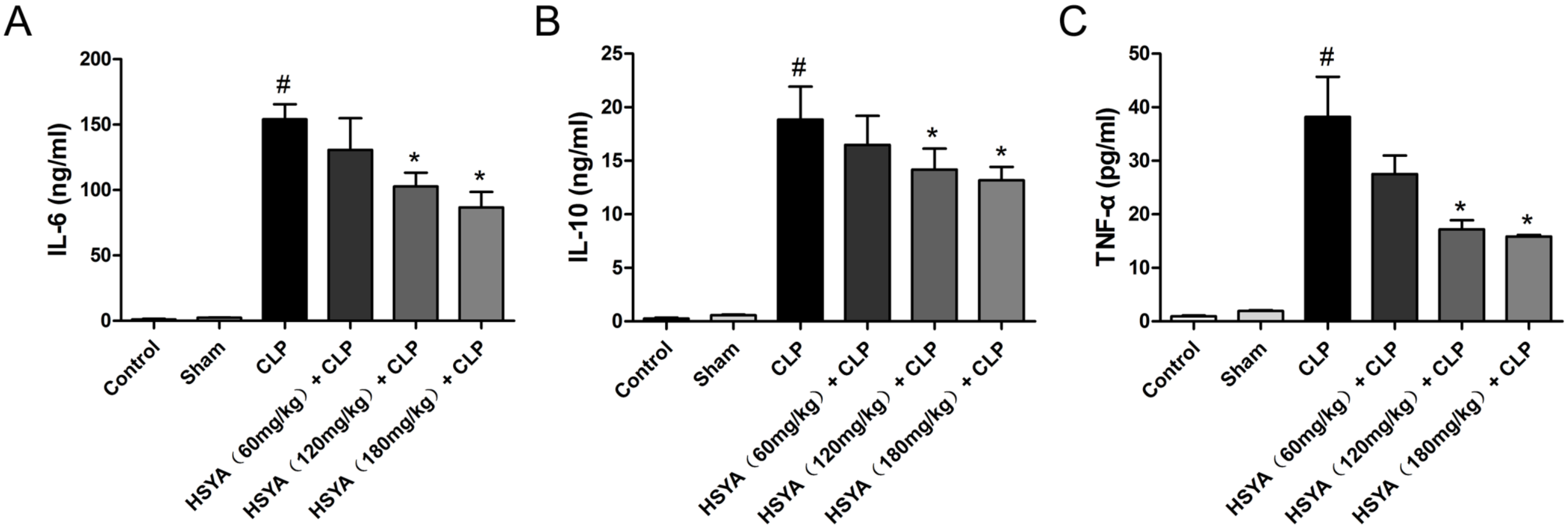

Supplementary Figure 1: Effect of HSYA on the production of cytokines in peripheral blood. Various doses of HSYA (60,120, 180mg/kg) were intravenously injected at 12h before the operation, and 0h and 12hafter CLP operation. Serum levels of (A) IL-6 (B) IL-10 and (C) TNF-alpha were detected by ELISA 24 h after CLP. Data are presented as mean ±SD of 6 animals per group. $^{\#}$Denotes significant differences (P<0.05) compared to control group.*Denotes significant differences (P< 0.05) compared to CLP group.
