# Supplementary material for: Hydroxysafflor Yellow A Attenuates the Apoptosis of Peripheral Blood CD4+ T Lymphocytes in a Murine Model of Sepsis
Source: Front Pharmacol. 2017 Sep 6;8:613. doi: 10.3389/fphar.2017.00613 (PMC5592278; doi:10.3389/fphar.2017.00613)
Supplement: Supplementary file 4 [file Image_2.PDF]

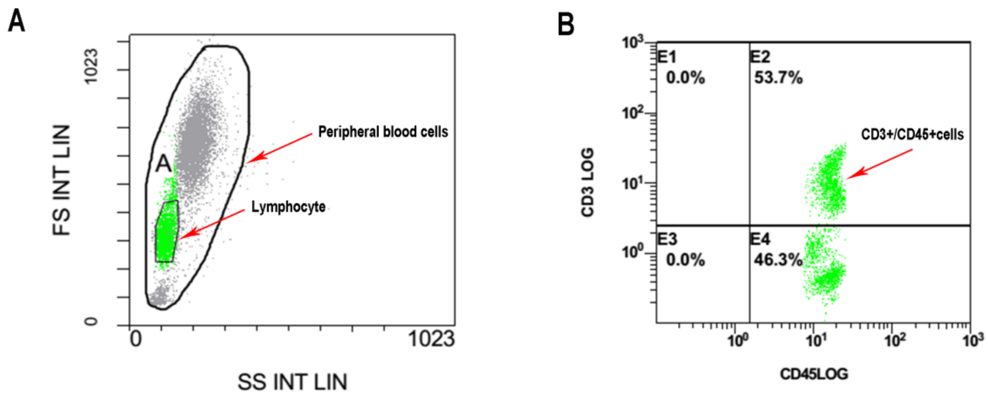

Supplementary Figure 2, Related to Figure 3: Detection of T lymphocyte population. Heparinized peripheral blood samples were collected. The erythrocytes were lysed with ammonium chloride (150mM) and washed twice in phosphate-buffered saline. The suspended cells were incubated for with anti-mouse CD3+and CD45+ antibodies. T lymphocyte population was characterized by CD3+/CD45+cells staining. Stained cells were detected using a FACSCalibur flow cytometry instrument. For each sample, 10,000 events were recorded, and analyzed by CellQuest software.
