# Supplementary material for: Hydroxysafflor Yellow A Attenuates the Apoptosis of Peripheral Blood CD4+ T Lymphocytes in a Murine Model of Sepsis
Source: Front Pharmacol. 2017 Sep 6;8:613. doi: 10.3389/fphar.2017.00613 (PMC5592278; doi:10.3389/fphar.2017.00613)
Supplement: Supplementary file 5 [file Image_3.pdf]

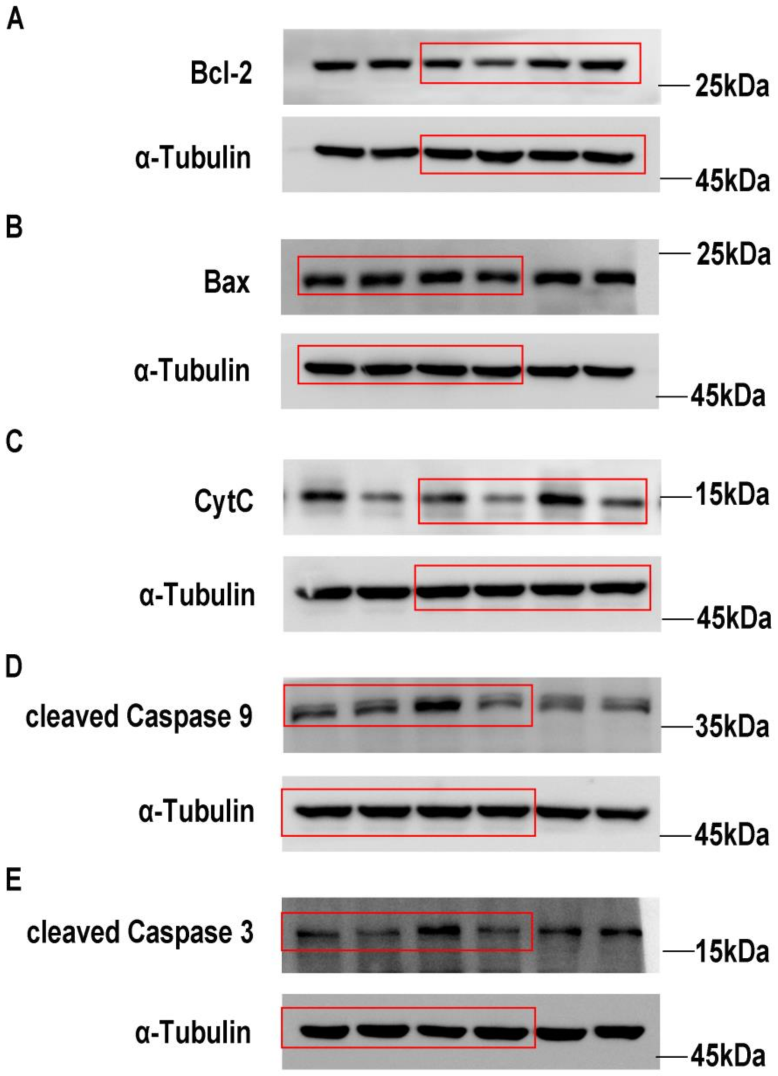

Supplementary Figure 3, uncropped full gel scan related to Figure 6: Effect of HSYA on the protein levels of Bcl-2, Cytc, Bax, cleaved caspase-3, and cleaved caspase-9 in $CD4^+$ T lymphocytes. HSYA (120 mg/kg) was intravenously injected at 12 h before the operation, 0 h and 12 h after CLP. Individual mouse sample was not sufficient for western blot analysis. Therefore, the $CD4^+$ T lymphocytes from each mouse in same group were pooled. Protein levels of Bcl-2 (A), Cytc (B), Bax (C), cleaved caspase-9 (D), and cleaved caspase-3 (E) were determined by western blot at 24 h after CLP. Alpha-tubulin was used as a loading control.
